# Duodenal Intussusception in an Adult With Situs Inversus Totalis

**DOI:** 10.7759/cureus.85984

**Published:** 2025-06-14

**Authors:** Diana Chien, Iman Rasheed

**Affiliations:** 1 Emergency Medicine, Eisenhower Health, Rancho Mirage, USA

**Keywords:** dextrocardia, duodenojejunal intussusception, intussusception, situs inversus, situs-inversus-totalis

## Abstract

Situs inversus totalis (SIT) is a rare congenital abnormality in which there is a mirror image of all thoracic and abdominal organs. Most individuals have no specific health issues. Their unique anatomy can pose challenges when they present to the emergency department with vague symptoms. The condition has been known to be associated with multiple anomalies, including Kartagener syndrome, congenital heart defects, asplenia, gastroschisis, and duodenal atresia, to name a few. However, it is rarely associated with intestinal obstruction. Duodenal intussusception occurs when a portion of the intestine telescopes into another portion of the duodenum and rarely occurs in adults. Here, we present a rare case of a 50-year-old female who presented to the emergency department with complaints of epigastric and left upper quadrant abdominal pain. Following thorough clinical and radiological evaluation, she was discovered to have SIT with evidence of duodenal intussusception. The patient was ultimately transferred to a tertiary center for evaluation by general surgery and gastroenterology and discharged for outpatient follow-up. To the best of our knowledge, this is the first reported case in the literature.

## Introduction

Situs inversus totalis (SIT) is a rare autosomal recessive genetic disorder in which there is a mirror image transposition of nearly all visceral organs in the thoracic and abdominal cavity. The incidence of this condition ranges from 1:10,000 to 1:20,000 [1,2]. Patients with SIT can suffer from a wide array of gastric abnormalities. Adult intussusception is rare and accounts for only 1% of all bowel obstructions and 5% of all cases of intussusception [3]. We present a rare case of duodenal intussusception in an adult with SIT.

## Case presentation

A 50-year-old female with a reported history of only diabetes presented to the emergency department with complaints of one day of left upper quadrant abdominal pain with associated non-bloody, non-bilious emesis. Prior to this, she reported that she was feeling well and had never experienced this type of pain before. She described it as a severe, stabbing-like pain that was non-radiating in nature. Past surgical history was significant for one prior cesarean section. On exam, she was well-appearing and was noted to be tender to palpation in her left upper quadrant with no signs of peritonitis. The rest of her exam was otherwise unremarkable. Differentials considered included pancreatitis, acute coronary syndrome (ACS), gastritis, pneumonia, splenic rupture, pyelonephritis, nephrolithiasis, bowel obstruction, and mesenteric ischemia.

**Figure 1:**
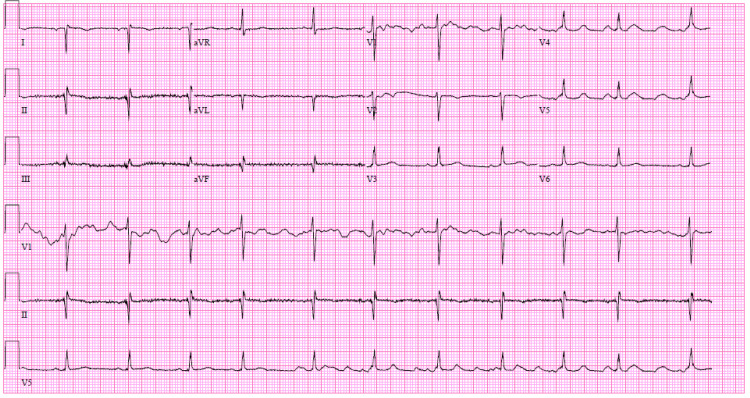
Normal sinus rhythm with occasional premature atrial complexes

**Figure 2:**
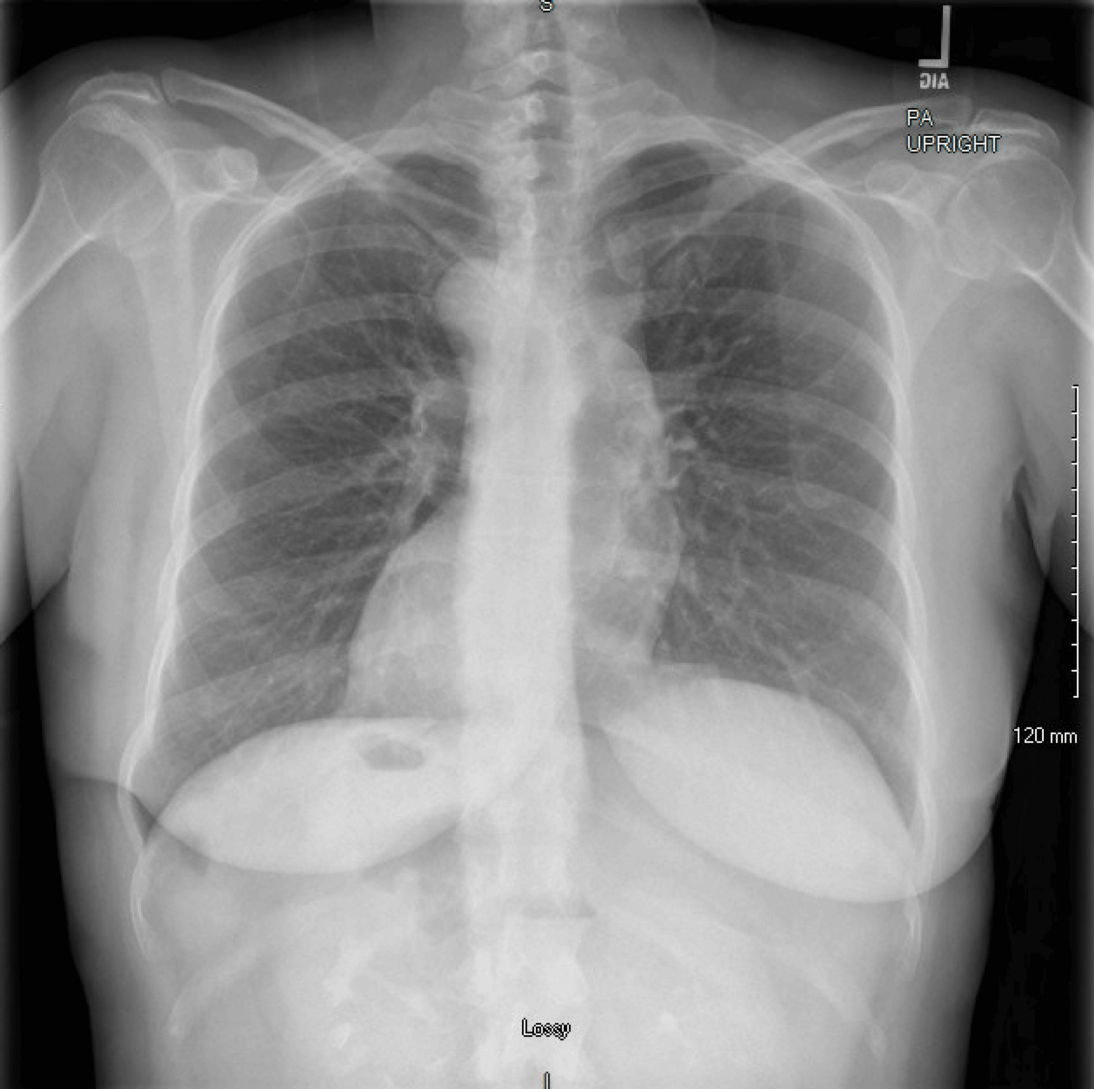
Chest X-ray demonstrating dextrocardia consistent with SIT. Note: The gastric bubble is located in the right upper quadrant of the abdomen as opposed to the left upper quadrant SIT: situs inversus totalis

Initial high-sensitivity troponin was noted to be <2 pg/mL (normal: <14 pg/mL), and electrocardiogram (ECG) showed evidence of normal sinus rhythm with no evidence of ischemia noted, making ACS unlikely (Figure 1). Lactate was normal at 1.6 mmol/L (normal: 0.5-2.2 mmol/L), making bowel ischemia less likely. Urinalysis showed no evidence of infection or hematuria. Prior to CT imaging, the patient received Maalox and viscous lidocaine for treatment of possible gastritis, with no improvement in symptoms. Comprehensive metabolic panel (CMP) showed no evidence of electrolyte abnormalities or renal impairment. Complete blood count (CBC) was significant for a mild leukocytosis with WBC 13.3 K/uL (normal: 3.8-10.8 k/uL) with a stable hemoglobin of 12.2 g/dL (normal: 12.0-16.0 g/dL). Lipase was noted to be mildly elevated to 245 U/L (normal: 11-82 U/L), which is just below 3x the upper limit of normal for our lab standards, making acute pancreatitis unlikely. A chest X-ray was first obtained, showing evidence of dextrocardia, with no evidence of pleural effusions, infiltrates, consolidations, or pneumothorax (Figure 2).

**Figure 3:**
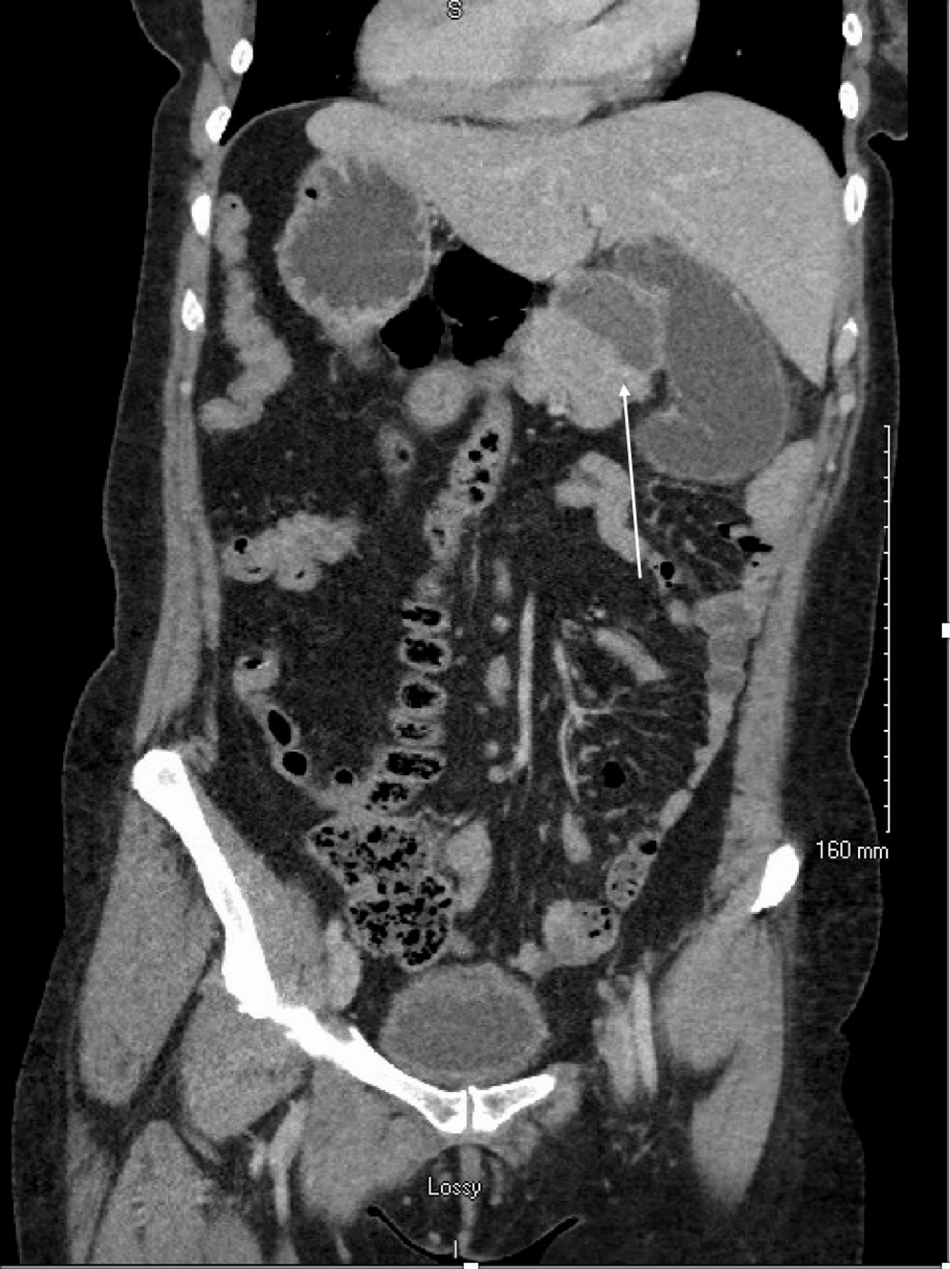
Coronal view of CT abdomen and pelvis with contrast consistent with SIT. The white arrow indicates duodenal intussusception. Note: The liver is located in the left upper quadrant SIT: situs inversus totalis

A CT abdomen/pelvis with and without intravenous (IV) contrast was then obtained, which showed evidence of SIT and intussusception at the level of the duodenum (Figure 3). The case was discussed with our general surgery and gastroenterology colleagues, who recommended transfer to a higher level of care given this patient's unique anatomy for operative repair. The patient was ultimately transferred to the emergency department of a tertiary care center. Following evaluation by appropriate specialists, the patient was discharged home for outpatient follow-up. The patient's case was ultimately lost at follow-up.

## Discussion

The term SIT originates from the Latin phrase “situs inversus viscerum.” It is often referred to as situs inversus with dextrocardia [4]. The heart is positioned in the right instead of the left hemithorax. The spleen, aorta, and stomach sit on the right, while the liver and gallbladder are seen on the left side of the abdomen [4,5]. Patients with this condition are typically unaware until they undergo some form of diagnostic imaging. They are usually asymptomatic and lead healthy lives with normal life expectancies. SIT can be associated with other congenital heart defects, GI abnormalities, or Kartagener syndrome, to name a few [5,6].

Intussusception occurs when one segment of the bowel telescopes into an adjacent segment, resulting in obstruction or ischemia. Children are most commonly affected, with a ratio of 20:1 [7]. Whereas in adults, it is much rarer and is found in less than one in 1,300 and represents only 1% of small bowel obstructions [8]. Recent literature has additionally described the rare occurrence of gastroduodenal intussusception in adults following gastric plication, a bariatric surgery [9].

To the best of our knowledge, the association between intussusception and SIT has not been established in the literature. There have been a few reported cases of ileocecal intussusception in SIT in both adults and children [2,10,11]. It has been previously hypothesized that anatomical variation of the ileocecal valve or decreased rigidity of the cecal wall in patients with SIT may make it more prone to prolapse and result in intussusception [2]. Perhaps this hypothesis could be extended to include the small intestine. However, to the best of our knowledge, there is no established literature to support this claim. There are articles reviewing independent cases of duodenal intussusception and SIT. However, there remains no discussion in the literature regarding the direct association of duodenal intussusception and SIT.

Abdominal CT appears to be the most sensitive diagnostic method in patients presenting with vague abdominal pain [11]. It is helpful in identifying the exact anatomical variations in both the chest and abdomen and can be used for surgical planning. Most cases of intussusception in adults are typically not clinically suspected. The appearance of CT images is similar to either a “target” or “sausage” mass, depending on the orientation of the CT beams [12]. Prompt consultation with general surgery and gastroenterology is crucial in managing these patients. Nearly 90% of cases of intussusception in adults have a pathological lead point, with gastric gastrointestinal stromal tumors (GISTs) being the most common etiology of gastroduodenal intussusception in adults [13,10]. Therefore, endoscopic evaluation is of equal importance as CT imaging in most cases. Surgical options for intussusception are not different in SIT patients. However, patients with SIT are at risk of diagnostic errors when initially presenting to the ER for abdominal complaints due to the unusual sites of pain.

## Conclusions

In conclusion, we reported a rare adult case of SIT with duodenal intussusception that has not been previously described. This patient’s transposed anatomy did not impact treatment options. However, care should be taken when making a diagnosis in this type of patient, given the unusual locations of their major organs. It is important for clinicians to maintain an index of suspicion for such conditions despite their rarity, particularly when patients present with vague complaints.
